# The importance of *CDC27* in cancer: molecular pathology and clinical aspects

**DOI:** 10.1186/s12935-021-01860-9

**Published:** 2021-03-09

**Authors:** Golnaz Ensieh Kazemi-Sefat, Mohammad Keramatipour, Saeed Talebi, Kaveh Kavousi, Roya Sajed, Nazanin Atieh Kazemi-Sefat, Kazem Mousavizadeh

**Affiliations:** 1 Department of Molecular Medicine, Faculty of Advanced Technologies in Medicine, Iran University of Medical Sciences, Shahid Hemmat Highway, P.O. Box: 14665-354, Tehran, 14496-14535 Iran; 2 Department of Medical Genetics, School of Medicine, Tehran University of Medical Sciences, Tehran, Iran; 3 Department of Medical Genetics, Faculty of Medicine, Iran University of Medical Sciences, Tehran, Iran; 4 Laboratory of Complex Biological Systems and Bioinformatics (CBB), Department of Bioinformatics, Institute of Biochemistry and Biophysics (IBB), University of Tehran, Tehran, Iran; 5 Department of Immunology, Faculty of Medical Sciences, Tarbiat Modares University, Tehran, Iran; 6 Cellular and Molecular Research Center, Faculty of Medicine, Iran University of Medical Sciences, Tehran, Iran

**Keywords:** Anaphase-Promoting complex–cyclosome, CDC27 protein, Cell cycle, Neoplasms, Upregulation, Downregulation, Tumorigenesis

## Abstract

**Background:**

CDC27 is one of the core components of Anaphase Promoting complex/cyclosome. The main role of this protein is defined at cellular division to control cell cycle transitions. Here we review the molecular aspects that may affect CDC27 regulation from cell cycle and mitosis to cancer pathogenesis and prognosis.

**Main text:**

It has been suggested that CDC27 may play either like a tumor suppressor gene or oncogene in different neoplasms. Divergent variations in *CDC27* DNA sequence and alterations in transcription of *CDC27* have been detected in different solid tumors and hematological malignancies. Elevated *CDC27* expression level may increase cell proliferation, invasiveness and metastasis in some malignancies. It has been proposed that *CDC27* upregulation may increase stemness in cancer stem cells. On the other hand, downregulation of *CDC27* may increase the cancer cell survival, decrease radiosensitivity and increase chemoresistancy. In addition, *CDC27* downregulation may stimulate efferocytosis and improve tumor microenvironment.

**Conclusion:**

CDC27 dysregulation, either increased or decreased activity, may aggravate neoplasms. CDC27 may be suggested as a prognostic biomarker in different malignancies.

**Supplementary Information:**

The online version contains supplementary material available at 10.1186/s12935-021-01860-9.

## Background

Neoplastic cells usually form by cellular transformation. This is a stepwise process which disturbs the harmonies between factors that regulate the cell cycle. The cell cycle transition, error-free chromosome duplication, segregation and finally exit of mitosis are ensured by the well timed activation of ubiquitination enzymes [1]. One of the most important ubiquitination enzymes is Anaphase-Promoting complex or cyclosome (APC/C). CDC27 is one of the core components of Anaphase Promoting complex/cyclosome.

Human studies about the role of CDC27 in cancer pathogenesis and its clinical importance are relatively few. Therefore, collecting the current knowledge and recent insights about this molecule in human may help better understanding its role in cancer and depicting the road map for future investigations.

### CDC27 in Anaphase Promoting complex (APC/C)

APC/C is composed of two specific sub-complexes: catalytic sub-complex and tetratricopeptide repeat (TPR) suprahelix sub-complex which the latter has scaffolding role [2, 3]. TPR suprahelix sub-complex orchestrates the position of substrate recognition sites in APC/C to perform ubiquitination [2, 4]. Human conserved canonical TPR subunits of APC/C including CDC27 (APC3), CDC16 (APC6) and CDC23 (APC8) consist of TPR motifs and have a quasi-symmetrical structure [4].

**Fig. 1:**
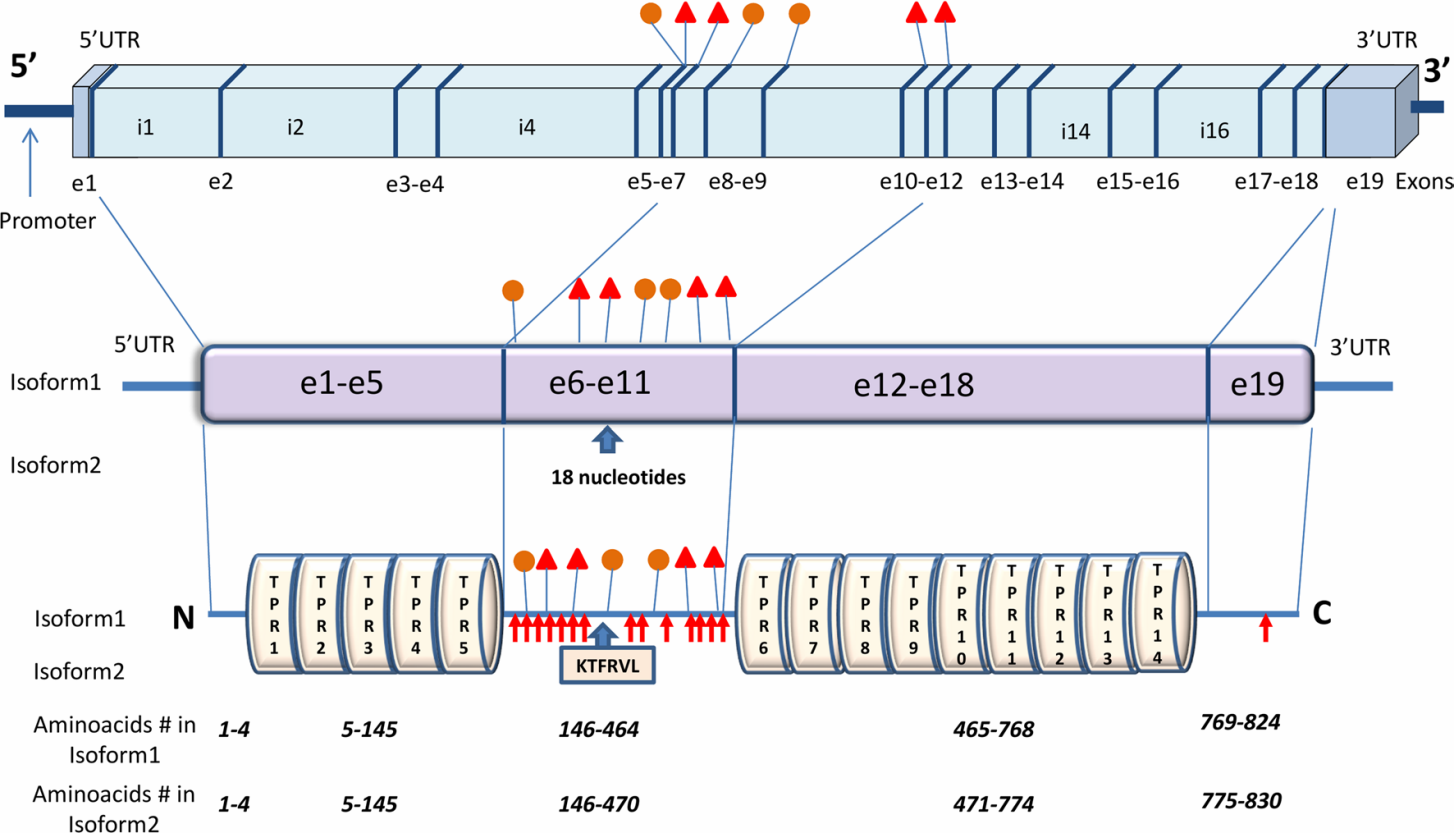
*CDC27* from DNA to protein. CDC27 longest isoform (Isoform 2) has 19 exons with 14 TPR motifs which are located in two TPR domains. Six aminoacids (KTFRL at the junction of exon 8 and exon 9) are the difference between the main CDC27 isoform with 824 aminoacids (Isoform 1) and the longest functional CDC27 isoform with 830 aminoacids (Isoform 2). Most pathologic germline (small circles) and somatic variants (small triangles) affect CDC27 amnioacids which are located between two TPR domains (exons 6–11). In addition, common phosphorylation sites in the CDC27 protein are serine and threonine amnioacids between two TPR domains (small arrows)

*CDC27* gene has 33 specific exons and undergoes alternative splicing that leads to multiple transcripts including 22 different mRNAs. Thirteen spliced mRNAs are supposed to translate to functional proteins. CDC27 main functional isoforms are coded by 19 exons and consist of 830 and 824 amino acids respectively with two TPR domains. The N-terminal domain has 5 TPR motifs and C-terminal domain has 9 motifs [5] (Fig. 1, Additional file 1: Table S1).

More than 16,494 *CDC27* variants have been recorded in human genome ensemble database, from which 1994 variants are on exons. Analyzing the distribution of variants on *CDC27* gene by calculating the variant density (frequency of variants per 100 bp sequence length) has shown that the 6th exon has the most variant density (Additional file 2: Figure S1, Additional file 1: Table S1, Fig. 1).

Eleven *CDC27* pseudogenes have been discovered on chromosomes 2, 14, 20, 21, 22 and Y [6]. The processed pseudogenes contain the complete cDNA sequence of *CDC27* from exon 3 to 14.

### Regulation of cell cycle by CDC27

The ubiquitin-mediated proteolysis is one of the main mechanisms of cell cycle regulation. The APC/C and the SCF (SKP1–CUL1–F-boxprotein) complexes are two ubiquitin ligases responsible for the specific ubiquitylation of many of cell cycle regulators [7]. Substrates from mid-M phase to the end of G1 phase are targeted by APC/C, whereas degradation of substrates from late G1 to early M phase is mediated by SCF ligases [8].

CDC27 can regulate mitosis and chromosome segregation by controlling APC/C activity in cyclins degradation. During mitosis, CDC27 accumulates in spindle microtubules, spindle poles and centrosomes [9] as well as chromosome arms and kinetochores [10].

In normal mitosis, interactions between some proteins and CDC27 may inhibit substrate binding to APC/C which regulates the timing of mitosis [10–12]. As an example, Early mitotic inhibitor 1 (Emi1) can inhibit APC activation. It has been revealed that CDC27 links Emi1 to APC/C core [13–15]. These negative regulations of APC/C prevent chromosome missegregation [16].

**Fig. 2:**
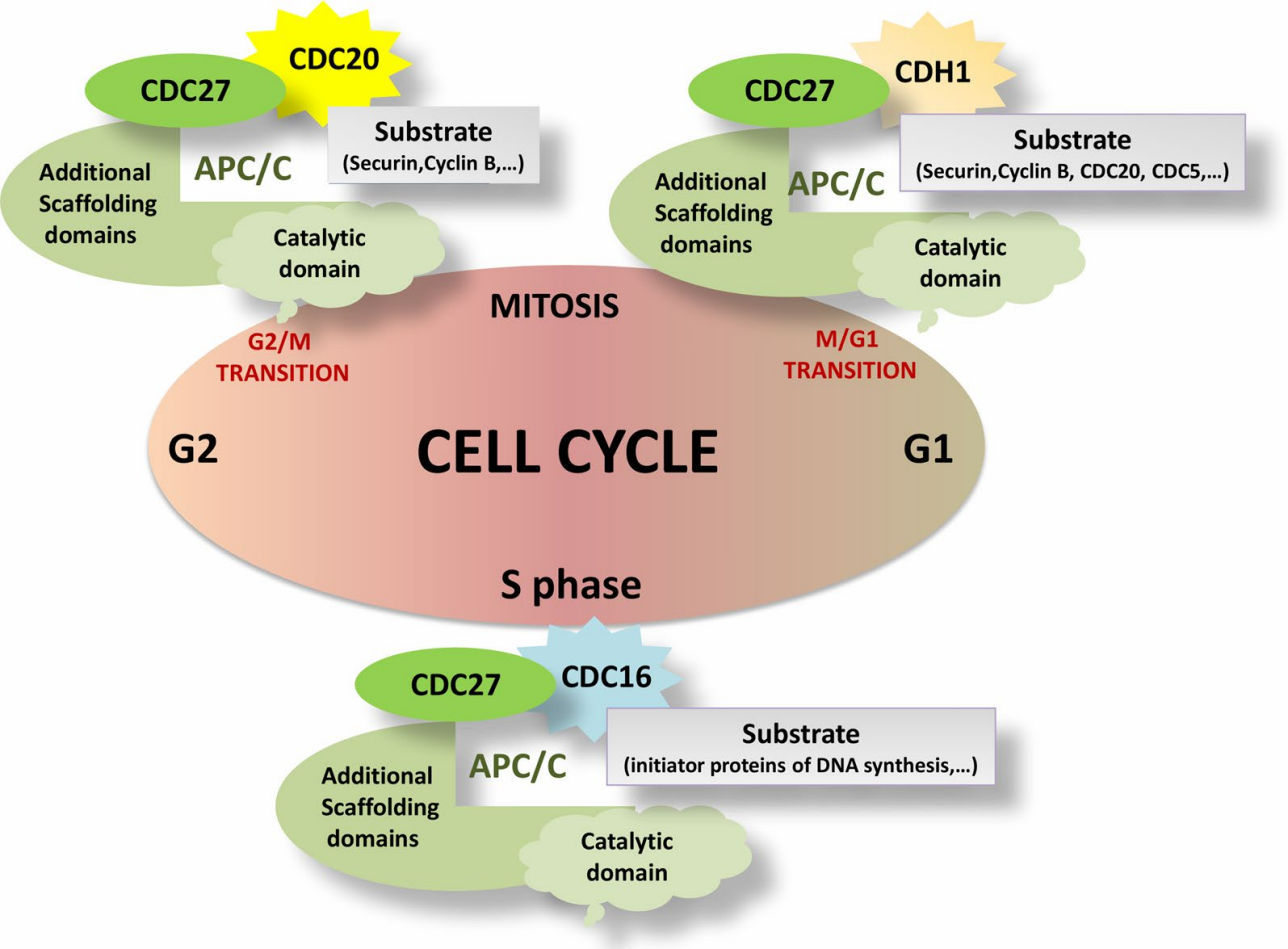
Cell cycle regulation by CDC27. At the G2/M transition CDC27 interacts with CDC20 and regulates proteolysis of proteins such as Securin and cyclin B. At the M/G1 transition, CDC27 interacts with CDH1. Therefore, CDC20, CDC5 and residual securin and cyclin B are targeted for degradation. At S phase, CDC27 and CDC16 by degradation of initiator proteins of DNA synthesis cause only one time DNA replication

CDC27 at the G2/M transition interacts with CDC20 (Fig. 2) and regulates ubiquitination and finally, proteolysis of proteins such as Securin and cyclin B to allow chromosome segregation [17, 18]. Securin is a regulator of cell cycle progression from metaphase to anaphase and in dephosphorylated form is one of the APC/C targets. When securin is triggered by APC/C, separase (that until then was inactivated by securin) degrades sister chromatid cohesions to synchronize faithful chromosomal segregation and ploidy stability [19].

CDC27 at the M/G1 transition [17] interacts with CDH1 (Fig. 2) and prepares all changes required for mitotic exit and transition into G1. Residual securin and cyclin B are targeted for degradation, as are CDC20 and CDC5, which put an end to the pattern of proteins required for mitosis [20].

It has been demonstrated that CDC27 expression modulates CDKN1A (p21: a cyclin-dependent kinase inhibitor in cell cycle) quantity and by this mechanism can control ID1 (as a regulator of G1/S transition during cell cycle) expression and G1 to S phase arrest/transition status [21–23].

CDC27 and CDC16 at S phase by degradation of the putative initiator proteins of DNA synthesis cause exclusively one time DNA replication per cell cycle in the yeast. Conversely, CDC27 or CDC16 mutants cause DNA to be over replicated [24]. This finding in human cell line models has not been investigated.

### Mechanisms suggested for the CDC27 regulation

Wide range of mechanisms could regulate activity of gene products at different levels [25]. Transcriptional and post-transcriptional regulations by several mechanisms are important pieces of caner genetics puzzle. Furthermore, gene product’s activity can be changed independently of RNA level due to translational and posttranslational modifications [26].

Here we mention the known evidence about CDC27 regulatory factors in different levels from transcription to post translational modifications that may reveal the big gap of knowledge in this field.

One of the most recognized transcription factors in the regulation of *CDC27* expression is C/EBPdelta [27]. C/EBPdelta (CCAAT/enhancer binding protein delta) is a transcription factor that may play role in many biological processes such as proliferation, differentiation, growth arrest, metabolism, motility, inflammation and other immune responses [28].

In post transcriptional stage, the most important microRNAs which may have regulatory role in *CDC27* are miR-218-2 and miR-27a. *CDC27* expression is a downstream target of miR-218-2 and miR-27a. Overexpression of miR-218-2 and miR-27a lead to *CDC27* downregulation [29, 30]. In the case of cancer, expression of various microRNAs is out of regulation and this directly affects the expression of key proteins during tumorigenesis [31].

In the translational step, heterogeneous nuclear ribonucleoprotein E1 (hnRNP E1) can regulate the translation of *CDC27* [32]. hnRNP E1 which is encoded by *PCBP1* (poly (rC)-binding protein gene) is an RNA binding protein that preferably binds to mRNA of genes which have tandem polycytosine motifs such as 3′-UTR of *CDC27* mRNA [32, 33].

The most important post translational modification in CDC27 is phosphrylation. CDC27 during mitosis in a phosphorylation-dependent manner can regulate APC/C activity. Various kinases such as casein kinase II (CKII), cyclin dependent kinase (CDK1) and Polo-like-kinase (PLK1) phosphorylate CDC27 especially at Threonine/Serine-Proline motifs [32, 34–37]. It has been suggested that TGF-β/Smad3 also can phosphorylate CDC27. *Cis*/*trans* isomerization of the phosphorylated CDC27 induces conformational changes that regulates CDC27 activity [38, 39].

Up regulation of CDC27 phosphorylation may increase its activity and sensitivity to mitotic checkpoints inhibition [20]. On the other hand, CDC27 dephosphorylation leads to increasing chromosomal instability and production of multinucleated cells.

It has been shown that different enzymes such as PP1 (Protein phosphatase1) dephosphorylate CDC27 [10]. Also, dissociation of CDC20 from CDC27 causes inhibition of CDC27 phosphorylation [40]. CDC27 dephosphorylation has an important role in TGF beta superfamily signaling [36, 41]. In addition, CDC27 dephosphorylation during mitosis leads to raised level of cyclin B and sister chromatid segregation prohibition [24].

### CDC27 in cancer

Generally, the malignancy related genes are classified as oncogenes (OG) or tumor suppressor (TSG) genes. Loss of function in TSGs and gain of function in OGs are the main suggested origins of tumorigenesis.

It has been suggested that CDC27 may play role either like a tumor suppressor gene or oncogene in different neoplasms [27, 42]. Divergent variations in *CDC27* DNA sequence and alterations in transcription of *CDC27* have been detected in different solid tumors and hematological malignancies.

Investigations about CDC27 level or sequence alterations in cancer, its involvement in processes such as apoptosis, epithelial to mesenchymal transition (EMT), stemness and efferocytosis and its association to cancer prognosis and treatment response may help to better understand cancer mechanisms and more efficiently manage malignancies in future.

### *CDC27* expression alterations and consequences in cancer

Alterations in CDC27 level and its suggestive effects have been described in different malignancies (Table 1). The vast majority of cancers indicated moderate to strong expression of CDC27 protein including colorectal, testis, thyroid, gastric cancers and lung adenocarcinoma [42–45]. It has been suggested that CDC27 may contribute in the activation of oncogenic pathways [42]. Therefore, upregulation of *CDC27* may enhance tumorigenesis.

**Table 1:** *CDC27* RNA expression changes in different neoplasms

Neoplasm	*CDC27* expression changes	Effect	References
Gastrointestinal cancers			
Tumoral rectum or colon tissue	Upregulation	CRC progression, patient’s survival	[23]
Breast cancer			
Human breast cancer tissues	Downregulation	Prognostic biomarker	[46]
Human breast cancer tissues	Upregulation	Potential to explain disease recurrence	[32]
Negative breast cancer cell lines (TNBC) (MDA-MB-435 and MDA-MB-231)	Downregulation	Radio-responsiveness	[30]
SCC of cervix			
Irradiated SiHa	Downregulation	Radio-responsiveness status and treatment failure	[47]
Glioma			
Human glia cell lines and glioma tissue	Downregulation	Chemoresistancy to β-lap	[29]
Lung cancer			
Lung adenocarcinoma	Upregulation	Cell cycle progression and tumor progression	[82]
Non-small cell lung carcinoma cell line (EGR1-overexpressing H1299 cells)	Downregulation	Tumor progression	[44]
Bladder cancer (BC)			
Cisplatin sensitive human BC cell line (T24) and cisplatin resistant BC cell line (T24R2)	Upregulation	Metastasis and recurrence of progressive BC	[83]

*IHC* Immunohistochemistry, *SCC* squamous cell carcinoma

CDC27 protein level in Non-hodgkin's lymphomas, prostate, glioma, breast cancer and renal cell carcinomas was very low or absent in some samples [45]. It has been proposed that *CDC27* may also act as a tumor suppressor gene [27]. Therefore, downregulation of *CDC27* or loss of function mutations in this gene may suppress its inhibitory effects on tumorigenesis.

Generally, *CDC27* overexpression leads to proliferation, tumor formation, migration and invasion, and knock down of *CDC27* gene inhibits these functions [43].

*CDC27* overexpression is in harmony with the tumor size, TNM (tumor (T), nodes (N), and metastases (M)) stage and distant metastasis in colorectal cancer (CRC). These findings reveal evidence for the relation of *CDC27* expression to tumor progression and poor patient’s survival [23].

Enhanced expression of CDC27 protein was in agreement with the relative expression of EMT biomarkers in gastric cancer tissues and was correlated with clinicopathological properties such as TNM stage and lymph node metastasis [43].

*CDC27* downregulation may play a crucial role in carcinogesis and drug resistance in glioma. Increased chemoresistancy of glioma cells to beta-lapachone (β-lap, as an antineoplastic agent) has been attributed to *CDC27* downregulation [29]. Downregulation of CDC27 in glioma, as a core component of APC/C, leads to inadequate ubiquitination of securin and various cyclins such as cyclinA1/2, cyclinB1, and cyclinD1 and their elevated expression at protein level. Consequently, delay in the G0/G1 phase transition occurs [29].

In breast cancer patients, immunohistochemial evaluations of CDC27 along with securin are the valuable prognostic biomarkers after lymph node examination. Downregulation of CDC27 combined with overexpression of securin have potential to predict 5-year overall survival of the patients [46]. In triple negative breast cancer cell lines, *CDC27* downregulation due to miR-27a overexpression is associated with poor response to radiotherapy [30].

In squamous cell cervix carcinoma, *CDC27* downregulation was correlated with a poor radio-responsiveness status and treatment failure. It has been shown that reduced expression of CDC27 in irradiated SiHa cell line (cervical cancer cell line) promotes cell survival. Conversely, higher expression of CDC27 in irradiated C33A (cervical cancer cell line) compared to SiHa cell line causes more cell death [47]. Irradiated CNE-1 cells (nasopharyngeal carcinoma cells) showed decreased level of CDC27, which suggested CDC27 is a part of mechanism of radiosensitivity [48].

It is important to consider that the vast majority of *CDC27* mRNA isoforms are not protein coding. Therefore, gene expression profile may suggest false positive in the upregulation results, and measurement of protein would be required to confirm CDC27 induction.

### CDC27 as a potential prognostic biomarker in cancer

CDC27 has been suggested as a prognostic biomarker in some cancers. Discovering potential biomarkers for assessing treatment response is a valuable indicator to differentiate between responder patients and those who are at risk of treatment failure. This information helps clinicians to change their strategies to other modalities in order to decrease the rate of toxicity and other side effects in clinical settings. Biomarkers are also useful for predicting the prognosis and survival [49].

Considering CDC27 as a potential prognostic biomarker, there are a number of obstacles that this potential biomarker must surpass before it can be applied in the clinic. CDC27 at mRNA level has heterogeneous behavior in some malignancies such as breast cancer. As it was mentioned, *CDC27* also has several isoforms at RNA and protein level. Therefore, additional studies may be necessary to determine which *CDC27* isoform in which tumor subtype has the strongest association with prognosis. Subsequent evaluations may involve validation of the original findings, including analytic validity, clinical validity, and clinical utility [50].

### *CDC27* germline and somatic variants

It has been proposed that some germline variants in *CDC27* may increase the susceptibility to cancers (Table 2). In breast cancer, homozygous or heterozygous rs11570443 (CT or CC) along with homozygous rs12601027 (TT) in *CDC27* have an association with the risk of cancer [51]. The rs11570443 is a Variant of Uncertain Significance (VUS) and is located upstream to the *CDC27* promoter which may have a regulatory function. However, the basic mechanism of this relationship is not clarified.

**Table 2:** The germline variants in *CDC27* associated to cancer susceptibility

Variant Coordinates (hg19)	dbSNP rs number	cDNA/Protein (NM_001114091)	Exon/intron position	Neoplasm	CGI classification	References
17-45267537-T-C	rs11570443	c.-999A>G	2 KB Upstream	Breast cancer	NA	[51]
17-45203468-C-T	rs12601027	c.2179−2142G>A	Intron 16	Breast cancer	Not protein-affecting	[51]
17-45257617-T-C	rs764792	c.103+1311A>G	Intron 2	Breast cancer	Not protein-affecting	[52]

*NA* not available, *VUS* Variant of Uncertain Significance

In another study which was about the role of 1084 functional germline variants in breast cancer, rs764792 in *CDC27* was correlated with the risk of high-grade breast cancer. However, this association was not significant after Bonferroni correction for multiple testing [52].

In addition to germline variants, several important functional somatic variants, including gene fusions, in *CDC27* have been reported in different malignancies. Most of them are between TPR5 and TPR6 motifs and some are classified as Tier 1 (Table 3).

**Table 3:** The somatic variants in *CDC27* associated to cancer progression

dbSNP rs number	cDNA/protein (NM_001114091)	Exon/intron position; TPR domain	Effect/Neoplasm	CGI classification	References
rs79201963	c.1549G>A;p.E517K	Exon12;TPR6	Tumorigenic roles in calcifying fibrous tumor of the pleura	Tier 1	[54]
rs77095606	c.644T>G;p.L215W	Exon6		Passenger	
rs199899451	c.505A>T;p.K169*	Exon6		Tier 1	
rs796969472	c.1801C>G;p.Q601E	Exon14;TPR8		NA	
rs775321736	c.1795G>A;p.A599T	Exon14;TPR8		Tier 1	
rs796538886	c.1459T>G;p.C487G	Exon12;TPR5		Tier 1	
rs7350889	c.794G>A;p.G265D	Exon7	Possible driver in a subset of sporadic vestibular schwannoma	Passenger	[53]
rs62077279	c.17A>G;p.E6G	Exon1;TPR1	Potential biomarker for Osteosarcoma	Tier 1	[56]
rs74628496	c.705T>C;p.I235I	Exon7	Tumorigenesis/molecular pathogenesis of colon cancer	NA	[59]
rs747953129	501A>G;p.T167T	Exon6		Polymorphism	
rs77467652	c.449C>A; p.S150Y	Exon4		Passenger	
rs193061947	c.704T>C; p.I235T	Exon7	Tumorigenesis/potential therapeutic targets in lung adenocarcinoma	NA	[60]
rs200611688	c.818C>G;p.A273G	Exon7		Polymorphism	
–	c.1034G>T;p.S345I	Exon9	Unknown roles in Adrenocortical carcinoma	Passenger	[66]
rs200940073	c.1541C>T;p.A514V	Exon12;TPR6		Tier 1	
rs202052665	c.1504T>C;p.Y502H	Exon12		Passenger	

*NA* not available, *VUS* Variant of Uncertain Significance

In sporadic vestibular schwannoma, the mutations in *CDC27* were clustered in cDNA position 754–796, corresponding to amino acids 252–266, between TPR5 and TPR6. This region is important for protein–protein interactions. In this tumor, *CDC27* variants (including p.G265D/rs7350889) were suggested as possible drivers of tumorigenesis [53].

The *CDC27* tumor-specific and coding somatic variants in calcifying fibrous tumor of the pleura were suggested to have a role in the tumorigenesis and molecular pathogenesis of this cancer [54]. The rs79201963, rs199899451, rs775321736 and rs796538886 variants are classified as Tier 1 using CGI prediction software tool (Cancer Genome Interpreter) [55]. In Osteosarcoma, (OS) pathogenic p.E6G *CDC27* Tier 1 somatic mutation was suggested to have an important role in regulating OS tumor cell division and suggested as potential biomarker for OS [56]. CDC27-OAT intrachromosomal fusion between *CDC27* as a cell cycle regulator and *OAT* (ornithine aminotransferase, an enzyme which produces ornithine) in aggressive prostate tumors was identified in some patients [57].

The importance of somatic variants of *CDC27* in the pathogenesis of cancer also have been suggested in other malignancies including the Follicular thyroid cancer [58], colon cancer [59], EGFR/KRAS/ALK-negative lung adenocarcinoma [60], Testicular germ cell tumors (TGCT) [61] and FLT3-ITD Sorafenib-Resistant Acute Myeloid Leukaemia. [62]. Several *CDC27* variants have been detected in Prostate cancer [63], Relapsed B-Cell Precursor Acute Lymphoblastic Leukemia [64], Duodenal adenocarcinoma [65] and Adrenocortical carcinoma [66], but their role in tumorigenesis is unknown. In a recent study in gastric cancer, it was shown that *CDC27* somatic mutations may be independently associated with peritoneal metastasis [67].

Usually, mutations in *CDC27* are loss of function mutations. Therefore, it is expected that the germline or the somatic mutations, including point mutations or deletions, in *CDC27* may decrease the activity or the level of this protein in the cell.

It is important to consider that the variants on *CDC27* pseudogenes may produce false positive findings, specifically in sequencing by next generation sequencing (NGS) technologies [68]. Therefore, all detected variants by whole exome sequencing (WES) or whole genome sequencing (WGS), specifically between exons 3 to 14, must be confirmed by sanger sequencing to exclude false positive variants. Generally, the variant density of reported variants on exons 3 to 14 is more than other *CDC27* exons which some may be due to the variants on the pseudogenes.

### *CDC27* variations and cancer mutational signatures

Mutagenesis due to cellular DNA damage and impaired repair mechanisms leave mutational signature or distinctive imprint on the cancer genome.

The somatic and germline variations in *CDC27* in esophageal squamous cell carcinomas (ESCC) were key regulators that have been suggested to affect mutational processes. In this malignancy, association between cancer signatures and germline polymorphisms of *CDC27* was significant. Somatic amplification of *CDC27* was correlated with lower rate of C > A substitution, the higher activity of Signature 1, and decreased activity of Signature 2. Patients with Signature 1 showed low burden of overall somatic single nucleotide variants (SNVs). Inversely, higher burden of SNVs was significantly associated with *CDC27* deletions [69].

### CDC27 may play role in apoptosis, stemness, efferocytosis and EMT

The involvement of the cell cycle proteins in apoptosis shed light on the potentially common pathways between apoptosis and mitosis. The role of CDC27 in apoptosis has been evaluated in the Jurkat cells (T cell leukemia cell line). The cleavage of CDC27 by caspase-3-like enzyme in the Fas signaling cascade subsequently avoids the ubiquitin ligase function of APC. Therefore, cyclins A and B stay intact and prevent cell cycle progression [70].

Apoptosis or programmed cell death is attenuated in cancers. Accordingly, cells become immortal and this is one of the most important underlying mechanisms of tumorigenesis, metastasis and drug resistance in cancer [71]. Therefore, suppression of apoptosis by aberrant signaling pathways in most cancers may lead to increased CDC27 activity and cell cycle progression.

Cancer stem cells (CSCs) are subpopulations of cells inside a tumor that possess characteristics related to normal stem cells such as self-renewal and differentiation, but they have some deviations from normal stem cells. For instance, CSCs have altered gene expression profiles and are resistant to conventional radiotherapy and chemotherapy. Therefore, CSCs are the origin of the cancer resistance and the reason of cancer recurrence. Targeting CSCs is one of the most important areas of cancer treatment, but due to lack of specific and sensitive biomarkers for these cells, usage of this strategy is challenging [72]. ID1 and p21 are two proteins that are correlated with self-renewal capacity of cancer stem cells. The possibility of relation between CDC27 expression in CSCs and stemness features in colorectal cancer has been evaluated. Results demonstrated that modulation of ID1 by CDC27 is one of the proposed mechanisms of p21 expression regulation. For that reason, CDC27 can be a potential therapeutic target of CSCs, but more investigations are needed to confirm this possibility [23].

Apart from CDC27 role in the cell cycle, footprint of this molecule in Efferocytosis has been traced. Efferocytosis is a term for phagocytosis of apoptotic cells [73]. Cancers use this mechanism to make the tumor microenvironment immunotolerant. Elmo1-Dock1-Rac pathway has a major role in efferocytosis. Elmo1 is a non-intrinsic catalytic protein which works as a coordinator between multiple proteins to make the appropriate interactions among them in different cellular processes. One of the proposed binding partners of Elmo1 is CDC27. The exact function of CDC27-Elmo1 is not well defined. It is possible that this interaction makes the Elmo1 ready to be ubiqunitated and degraded by APC via Proteasome [74].

Epithelial to mesenchymal transition (EMT) has a major role in cancer metastasis. EMT explains how cells dedifferentiate and achieve increased invasive and migratory properties [75]. CDC27 by modulating ID1 can downregulate the expression of epithelial markers (ZO-1 and Ecadherin), and adversely upregulate mesenchymal markers (ZEB1 and Snail) to promote metastasis in colorectal cancer cell lines (HCT116 and DLD1). This claim has been confirmed in xenograft mouse model [42]. Furthermore, in gastric cancer tissues, enhanced expression of CDC27 protein was in harmony with the relative expression of EMT biomarkers (E-cadherin, Vimentin and Twist) [43].

Hence, diminished CDC27 activity may improve efferocytosis. Increment in efferocytosis may lead to increased cancer cell survival, decreased radiosensitivity and increased chemoresistancy. On the other hand, raised CDC27 activity may promote stemness and EMT which may increase tumorigenesis and metastasis.

### Therapeutic interventions related to *CDC27*

The natural and chemical molecules have been vastly studied for finding resources for prevention/treatment of malignancies by different proposed mechanism of actions. For instance, Resveratrol, a natural phytoestrogen, in A549 cells (lung cancer cells) inhibits cells proliferation. Resveratrol downregulates gene and protein expression of CDC27, and these alterations of expression along with some other mechanisms trap cells in G1/S or G2/M phases of cell cycle [76].

Also, in two distinct studies the interaction between Curcumin and CDC27 in various cancer cell types such as medulloblastoma and oral cancer cells has been investigated. Curcumin attachment preferentially to phosphorylated form of CDC27 as a core component of APC/C cross links the dimerized CDC27 molecules, interferes with its function, and eventually leads to cell cycle arrest at G2/M phase. CDC27, especially in phosphorylated form, has been suggested as a biomarker for the evaluation of anti-cancer effects of curcumin [77, 78].

Treating breast cancer cells (MCF10-F) with Etodolac (a member of NSAIDs) alters the expression profile of many genes including *CDC27.* It has been suggested that NSAIDs arrest the cell cycle at G1 and avoid cell cycle advancement as well as DNA synthesis. This may be one of the explanations for Etodolac inducing cancer cell death [79].

Finally, after treatment of ovarian cancer cells with Eribulin and Paclitaxel (two anti-cancer drugs), expression of *CDC27* was decreased at both mRNA and protein level. Therefore, *CDC27* as an oncogene, is one of the related genes proposed for growth inhibiting action of Eribulin and Paclitaxel on ovarian cancer cells [80]. More investigations are needed to discover the precise underlying molecular mechanisms.

## Conclusion

**Fig. 3:**
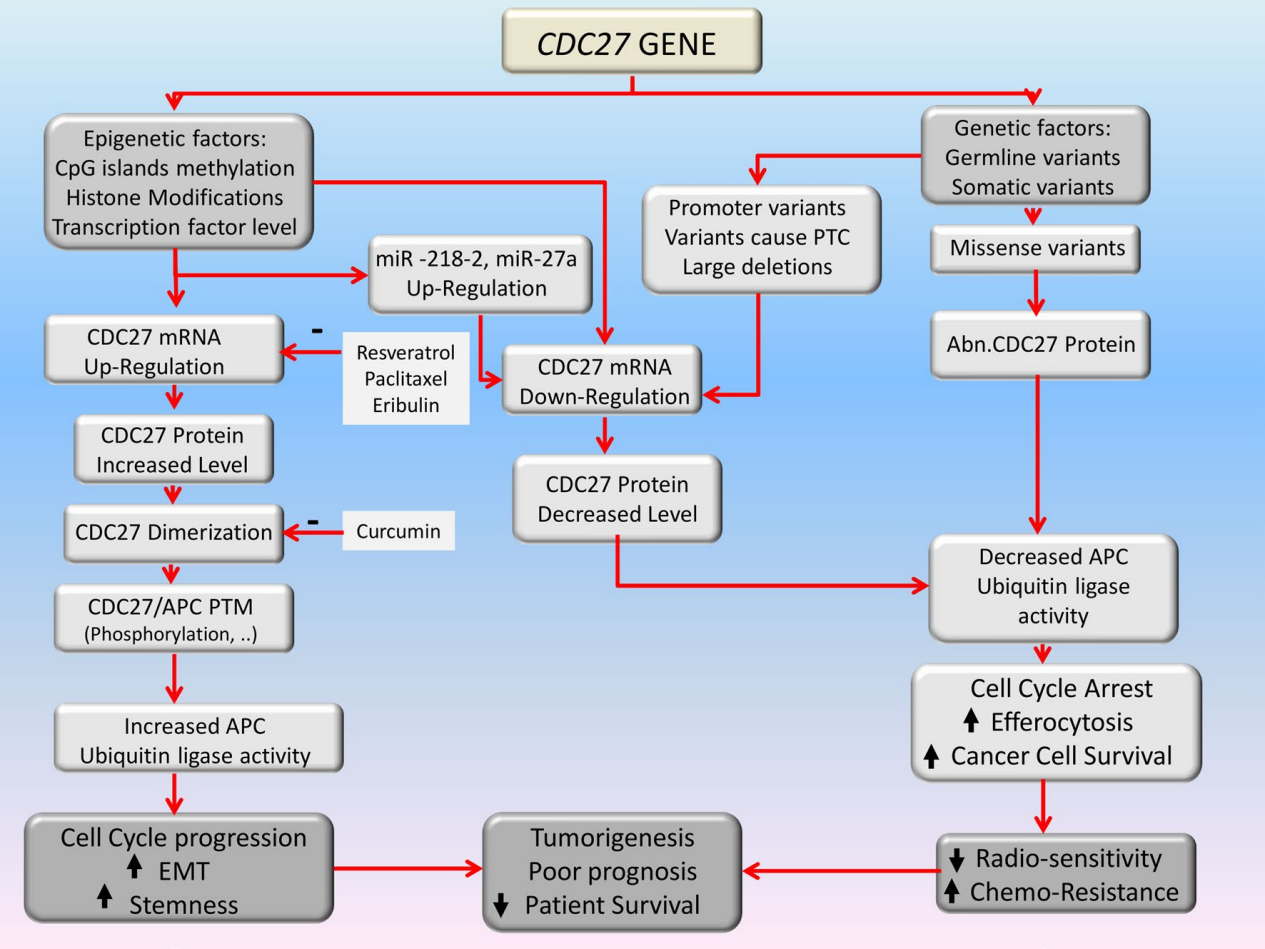
Probable mechanisms related to the *CDC27* dysregulation in cancer and their effects on tumorigenesis and prognosis. The mechanisms which alter *CDC27* gene function in cancer are classified as genetic and epigenetic events at different levels of DNA, RNA and protein. In genetic events, missense mutations usually change the amnioacid sequence of protein which subsequently leads to an abnormal protein function. Large deletions and promoter mutation decrease the level of transcription. In mutations with Premature Termination Codon (PTC), usually the level of mRNA decreases by Nonsense Mediated Decay (NMD). Epigenetic events including CpG island methylation changes, Histone modifications, gene specific miRNAs and alterations in transcription factor levels can change the level of gene expression. Collectively increased CDC27 is associated to cell cycle progression, EMT and stemness which may increase tumorigenesis. On the other hand, decreased activity of CDC27 is associated to cell cycle arrest, increased efferocytosis and cancer cell survival which may lead to increased chemoresistancy and decreased radiosensitivity. Therefore, dysregulation of CDC27 activity (both increased and decreased) is related to tumorigenesis and poor prognosis and may decrease patient’s survival

Cell division, genome stability, differentiation, carcinogenesis, autophagy, cell death, as well as energy metabolism can be regulated by APC/C [81]. Most of these functions which are important in cancer pathogenesis may be regulated by CDC27 subunit in APC/C. Alterations in *CDC27* at the DNA, RNA and protein levels and post translational modifications may affect cell division. Accordingly, have divergent effects on tumorigenesis, response to treatment and eventually, prognosis and survival of patients (Fig. 3).

At DNA level, both germline and somatic variants may affect CDC27 function and have a role in tumorigenesis. *CDC27* dysregulation at RNA level, either upregulation or downregulation, may affect the patient’s survival and prognosis. More investigations are needed to discover the exact role of CDC27 in cancer. The advancement of new technologies has made it possible to evaluate the altered function of the product of this gene at single cell level and even at the serial time points of diverse phases of cell cycle.

## Supplementary Information

**Additional file 1: Table S1.** Protein sequence in exons and TPR motifs of CDC27.* CDC27* has 19 exons with 14 TPR motifs which are located in two TPR domains. Aminoacid numbers are according to the longest isoform with 830 aminoacids. Six yellow highlighted aminoacids in brackets (at the junction of exon 8 and exon 9) are the difference between the main* CDC27* isoform with 824 aminoacids and the second important functional* CDC27* isoform with 830 aminoacids. Different exons are colored alternately blue and black. Red colored aminoacids at exon junctions are coded by a codon which has nucleotides on both exons. Purple highlighted aminoacids are common phosphorylation sites in the CDC27 protein which all of them are located between two TPR domains. The structure of the APC3 is consisted of 14 units of the TPR motif, which are organised as follows: dimerization domain (TPR 1 to TPR 7), IR tail binding domain (TPR 8 to TPR 11), and C-terminal domain (TPR 12 to TPR 14).

**Additional file 2: Figure S1.** The frequency of potentially somatic and germline variants at exons and introns in* CDC27* gene. The frequency is calculated as the number of variants per 100 bases in each exon or intron (Number of variants is divided to the exon or intron length and then multiplied by 100). About 588* CDC27* variants were listed in COSMIC (554 variants on exons). This means that potentially somatic cancer variants may compose more than 25% of detected variants on* CDC27* exons.

## Data Availability

The datasets generated and/or analysed during the current study are available in the [CGI] repository, [https://www.cancergenomeinterpreter.org]. The datasets generated and/or analysed during the current study are available in the [AceView] repository, [https://www.ncbi.nlm.nih.gov/IEB/Research/Acembly/av.cgi?db=human&c=Gene&l=CDC27]. The datasets generated and/or analysed during the current study are available in the [The Human Protein Atlas] repository, [https://www.proteinatlas.org/ENSG00000004897-CDC27/pathology.]

## Abbreviations

APC/C: Anaphase-Promoting complex or cyclosome
TPR: Tetratricopeptide repeat
Emi1: Early mitotic inhibitor 1
OG: Oncogenes
TSG: Tumor suppressor
EMT: Epithelial to mesenchymal transition
CRC: Colorectal cancer
β-lap: Beta-lapachone
C/EBPdelta: CCAAT/enhancer binding protein delta
HNRNP E1: Heterogeneous nuclear ribonucleoprotein E1
CKII: Casein Kinase II
CDK1: Cyclin dependent kinase
PLK1: Polo-like-kinase
PIN1: Peptidyl-prolyl *cis*–*trans* isomerase NIMA-interacting 1
PP1: Protein phosphatase 1
WES: Whole exome sequencing
CGI: Cancer Genome Interpreter
TGCT: Testicular germ cell tumors
OS: Osteosarcoma
OAT: Ornithine aminotransferase
ESCC: Esophageal squamous cell carcinomas
SNVs: Single nucleotide variants
CSCs: Cancer stem cells
PTC: Premature Termination Codon
NMD: Nonsense Mediated Decay
TNM: Tumor (T), nodes (N), and metastases (M)

## References

[1] Wickliffe K, Williamson A, Jin L, Rape M (2009). The multiple layers of ubiquitin-dependent cell cycle control. Chem Rev.

[2] Das AK, Cohen PW, Barford D (1998). The structure of the tetratricopeptide repeats of protein phosphatase 5: implications for TPR-mediated protein-protein interactions. EMBO J.

[3] Zhang Z, Yang J, Kong EH, Chao WC, Morris EP, da Fonseca PC (2013). Recombinant expression, reconstitution and structure of human anaphase-promoting complex (APC/C). Biochem J.

[4] Schreiber A, Stengel F, Zhang Z, Enchev RI, Kong EH, Morris EP (2011). Structural basis for the subunit assembly of the anaphase-promoting complex. Nature.

[5] https://www.ncbi.nlm.nih.gov/IEB/Research/Acembly/av.cgi?db=human&c=Gene&l=CDC27. Accessed 12 Dec 2019.

[6] https://www.ncbi.nlm.nih.gov/IEB/Research/Acembly/av.cgi?db=human&c=Gene&l=CDC27. Accessed 21 Jan 2021.

[7] Nakayama KI, Nakayama K (2006). Ubiquitin ligases: cell-cycle control and cancer. Nat Rev Cancer.

[8] Teixeira LK, Reed SI (2013). Ubiquitin ligases and cell cycle control. Annu Rev Biochem.

[9] Tugendreich S, Tomkiel J, Earnshaw W, Hieter P (1995). CDC27Hs colocalizes with CDC16Hs to the centrosome and mitotic spindle and is essential for the metaphase to anaphase transition. Cell.

[10] Topper LM, Campbell MS, Tugendreich S, Daum JR, Burke DJ, Hieter P (2002). The dephosphorylated form of the anaphase-promoting complex protein Cdc27/Apc3 concentrates on kinetochores and chromosome arms in mitosis. Cell Cycle.

[11] Kallio M, Weinstein J, Daum JR, Burke DJ, Gorbsky GJ (1998). Mammalian p55CDC mediates association of the spindle checkpoint protein Mad2 with the cyclosome/anaphase-promoting complex, and is involved in regulating anaphase onset and late mitotic events. J Cell Biol.

[12] Lee J, Moon B, Lee DH, Lee G, Park D (2016). Identification of a novel protein interaction between Elmo1 and Cdc27. Biochem Biophys Res Commun.

[13] Chang L, Zhang Z, Yang J, McLaughlin SH, Barford D (2015). Atomic structure of the APC/C and its mechanism of protein ubiquitination. Nature.

[14] Miller JJ, Summers MK, Hansen DV, Nachury MV, Lehman NL, Loktev A (2006). Emi1 stably binds and inhibits the anaphase-promoting complex/cyclosome as a pseudosubstrate inhibitor. Genes Dev.

[15] Pesin JA, Orr-Weaver TL (2008). Regulation of APC/C activators in mitosis and meiosis. Annu Rev Cell Dev Biol.

[16] Wassmann K, Benezra R (1998). Mad2 transiently associates with an APC/p55Cdc complex during mitosis. Proc Natl Acad Sci USA.

[17] Harkness TAA (2018). Activating the Anaphase promoting complex to enhance genomic stability and prolong lifespan. Int J Mol Sci.

[18] Prinz S, Hwang ES, Visintin R, Amon A (1998). The regulation of Cdc20 proteolysis reveals a role for APC components Cdc23 and Cdc27 during S phase and early mitosis. Curr Biol.

[19] Holt LJ, Krutchinsky AN, Morgan DO (2008). Positive feedback sharpens the anaphase switch. Nature.

[20] Yen TJ (2002). The complexity of APC/C regulation: location, location, location. Cell Cycle.

[21] Abbas T, Dutta A (2009). p21 in cancer: intricate networks and multiple activities. Nat Rev Cancer.

[22] Alani RM, Young AZ, Shifflett CB (2001). Id1 regulation of cellular senescence through transcriptional repression of p16/Ink4a. Proc Natl Acad Sci USA.

[23] Qiu L, Wu J, Pan C, Tan X, Lin J, Liu R (2016). Downregulation of CDC27 inhibits the proliferation of colorectal cancer cells via the accumulation of p21Cip1/Waf1. Cell Death Dis.

[24] Heichman KA, Roberts JM (1996). The yeast CDC16 and CDC27 genes restrict DNA replication to once per cell cycle. Cell.

[25] Holliday R, Jeggo PA (1985). Mechanisms for changing gene expression and their possible relationship to carcinogenesis. Cancer Surv.

[26] Sager R (1997). Expression genetics in cancer: shifting the focus from DNA to RNA. Proc Natl Acad Sci USA.

[27] Pawar SA, Sarkar TR, Balamurugan K, Sharan S, Wang J, Zhang Y (2010). C/EBP{delta} targets cyclin D1 for proteasome-mediated degradation via induction of CDC27/APC3 expression. Proc Natl Acad Sci USA.

[28] Ko CY, Chang WC, Wang JM (2015). Biological roles of CCAAT/Enhancer-binding protein delta during inflammation. J Biomed Sci.

[29] Feng Z, Zhang L, Zhou J, Zhou S, Li L, Guo X (2017). mir-218-2 promotes glioblastomas growth, invasion and drug resistance by targeting CDC27. Oncotarget.

[30] Ren YQ, Fu F, Han J (2015). MiR-27a modulates radiosensitivity of triple-negative breast cancer (TNBC) cells by targeting CDC27. Med Sci Monit.

[31] Ghasabi M, Mansoori B, Mohammadi A, Duijf PH, Shomali N, Shirafkan N (2019). MicroRNAs in cancer drug resistance: basic evidence and clinical applications. J Cell Physiol.

[32] Link LA, Howley BV, Hussey GS, Howe PH (2016). PCBP1/HNRNP E1 protects chromosomal integrity by translational regulation of CDC27. Mol Cancer Res.

[33] Du Z, Fenn S, Tjhen R, James TL (2008). Structure of a construct of a human poly(C)-binding protein containing the first and second KH domains reveals insights into its regulatory mechanisms. J Biol Chem.

[34] Kotani S, Tugendreich S, Fujii M, Jorgensen PM, Watanabe N, Hoog C (1998). PKA and MPF-activated polo-like kinase regulate anaphase-promoting complex activity and mitosis progression. Mol Cell.

[35] Singh N, Wiltshire TD, Thompson JR, Mer G, Couch FJ (2012). Molecular basis for the association of microcephalin (MCPH1) protein with the cell division cycle protein 27 (Cdc27) subunit of the anaphase-promoting complex. J Biol Chem.

[36] Zhang L, Fujita T, Wu G, Xiao X, Wan Y (2011). Phosphorylation of the anaphase-promoting complex/Cdc27 is involved in TGF-beta signaling. J Biol Chem.

[37] Enserink JM, Kolodner RD (2010). An overview of Cdk1-controlled targets and processes. Cell Div.

[38] Lu KP, Finn G, Lee TH, Nicholson LK (2007). Prolyl cis-trans isomerization as a molecular timer. Nat Chem Biol.

[39] Shen M, Stukenberg PT, Kirschner MW, Lu KP (1998). The essential mitotic peptidyl-prolyl isomerase Pin1 binds and regulates mitosis-specific phosphoproteins. Genes Dev.

[40] Lee YJ, Lee HJ, Lee JS, Jeoung D, Kang CM, Bae S (2008). A novel function for HSF1-induced mitotic exit failure and genomic instability through direct interaction between HSF1 and Cdc20. Oncogene.

[41] Stroschein SL, Bonni S, Wrana JL, Luo K (2001). Smad3 recruits the anaphase-promoting complex for ubiquitination and degradation of SnoN. Genes Dev.

[42] Qiu L, Tan X, Lin J, Liu RY, Chen S, Geng R (2017). CDC27 induces metastasis and invasion in colorectal cancer via the promotion of epithelial-to-mesenchymal transition. J Cancer.

[43] Xin Y, Ning S, Zhang L, Cui M (2018). CDC27 facilitates gastric cancer cell proliferation, invasion and metastasis via twist-induced epithelial-mesenchymal transition. Cell Physiol Biochem.

[44] Zhang H, Chen X, Wang J, Guang W, Han W, Zhang H (2014). EGR1 decreases the malignancy of human non-small cell lung carcinoma by regulating KRT18 expression. Sci Rep.

[45] https://www.proteinatlas.org/ENSG00000004897-CDC27/pathology. Accessed 12 Dec 2019.

[46] Talvinen K, Karra H, Pitkanen R, Ahonen I, Nykanen M, Lintunen M (2013). Low cdc27 and high securin expression predict short survival for breast cancer patients. APMIS Acta Pathol Microbiol Immunol Scand.

[47] Rajkumar T, Gopal G, Selvaluxmi G, Rajalekshmy KR (2005). CDC27 protein is involved in radiation response in squamous cell cervix carcinoma. Indian J Biochem Biophys.

[48] Wang C, Su Z, Hou H, Li D, Pan Z, Tian W (2017). Inhibition of anaphase-promoting complex by silence APC/C(Cdh1) to enhance radiosensitivity of nasopharyngeal carcinoma cells. J Cell Biochem.

[49] Amin S, Bathe OF (2016). Response biomarkers: re-envisioning the approach to tailoring drug therapy for cancer. BMC Cancer.

[50] Henry NL, Hayes DF (2012). Cancer biomarkers. Mol Oncol.

[51] Guo H, Chen W, Ming J, Zhong R, Yi P, Zhu B (2015). Association between polymorphisms in cdc27 and breast cancer in a Chinese population. Tumour Biol.

[52] Stevens KN, Wang X, Fredericksen Z, Pankratz VS, Cerhan J, Vachon CM (2011). Evaluation of associations between common variation in mitotic regulatory pathways and risk of overall and high grade breast cancer. Breast Cancer Res Treat.

[53] Havik AL, Bruland O, Myrseth E, Miletic H, Aarhus M, Knappskog PM (2018). Genetic landscape of sporadic vestibular schwannoma. J Neurosurg.

[54] Mehrad M, LaFramboise WA, Lyons MA, Trejo Bittar HE, Yousem SA (2018). Whole-exome sequencing identifies unique mutations and copy number losses in calcifying fibrous tumor of the pleura: report of 3 cases and review of the literature. Hum Pathol.

[55] https://www.cancergenomeinterpreter.org. Accessed 12 Dec 2019.

[56] Reimann E, Koks S, Ho XD, Maasalu K, Martson A (2014). Whole exome sequencing of a single osteosarcoma case–integrative analysis with whole transcriptome RNA-seq data. Hum Genomics.

[57] Lindquist KJ, Paris PL, Hoffmann TJ, Cardin NJ, Kazma R, Mefford JA (2016). Mutational landscape of aggressive prostate tumors in African American men. Cancer Res.

[58] Erinjeri NJ, Nicolson NG, Deyholos C, Korah R, Carling T (2018). Whole-exome sequencing identifies two discrete druggable signaling pathways in follicular thyroid cancer. J Am Coll Surg.

[59] Yu C, Yu J, Yao X, Wu WK, Lu Y, Tang S (2014). Discovery of biclonal origin and a novel oncogene SLC12A5 in colon cancer by single-cell sequencing. Cell Res.

[60] Ahn JW, Kim HS, Yoon JK, Jang H, Han SM, Eun S (2014). Identification of somatic mutations in EGFR/KRAS/ALK-negative lung adenocarcinoma in never-smokers. Genome Med.

[61] Litchfield K, Summersgill B, Yost S, Sultana R, Labreche K, Dudakia D (2015). Whole-exome sequencing reveals the mutational spectrum of testicular germ cell tumours. Nat Commun.

[62] Lam SSY, Cher CY, Ng NKL, Man CH, Leung AYH (2015). Whole exome sequencing of FLT3-ITD sorafenib-resistant acute myeloid leukaemia. Blood.

[63] Lindberg J, Mills IG, Klevebring D, Liu W, Neiman M, Xu J (2013). The mitochondrial and autosomal mutation landscapes of prostate cancer. Eur Urol.

[64] Schroeder MP, Neumann M, Eckert C, Bastian L, James AR, Gökbuget N (2016). Multi-genomics of relapsed B-cell precursor acute lymphoblastic leukemia reveals three distinct genetic clusters characterized by different alterations. Blood.

[65] Yuan W, Zhang Z, Dai B, Wei Q, Liu J, Liu Y (2016). Whole-exome sequencing of duodenal adenocarcinoma identifies recurrent Wnt/beta-catenin signaling pathway mutations. Cancer.

[66] Juhlin CC, Goh G, Healy JM, Fonseca AL, Scholl UI, Stenman A (2015). Whole-exome sequencing characterizes the landscape of somatic mutations and copy number alterations in adrenocortical carcinoma. J Clin Endocrinol Metab.

[67] Wu R, Li Q, Wu F, Shi C, Chen Q (2020). Comprehensive analysis of CDC27 related to peritoneal metastasis by whole exome sequencing in gastric cancer. Onco Targets Ther.

[68] Knies K, Schuster B, Ameziane N, Rooimans M, Bettecken T, de Winter J (2012). Genotyping of fanconi anemia patients by whole exome sequencing: advantages and challenges. PLoS ONE.

[69] Guo J, Huang J, Zhou Y, Zhou Y, Yu L, Li H (2018). Germline and somatic variations influence the somatic mutational signatures of esophageal squamous cell carcinomas in a Chinese population. BMC Genomics.

[70] Zhou BB, Li H, Yuan J, Kirschner MW (1998). Caspase-dependent activation of cyclin-dependent kinases during Fas-induced apoptosis in Jurkat cells. Proc Natl Acad Sci USA.

[71] Wong RS (2011). Apoptosis in cancer: from pathogenesis to treatment. J Exp Clin Cancer Res.

[72] Yu Z, Pestell TG, Lisanti MP, Pestell RG (2012). Cancer stem cells. Int J Biochem Cell Biol.

[73] Werfel TA, Cook RS (2018). Efferocytosis in the tumor microenvironment. Semin Immunopathol.

[74] Makino Y, Tsuda M, Ohba Y, Nishihara H, Sawa H, Nagashima K (2015). Tyr724 phosphorylation of ELMO1 by Src is involved in cell spreading and migration via Rac1 activation. Cell Commun Signal.

[75] Karlsson MC, Gonzalez SF, Welin J, Fuxe J (2017). Epithelial–mesenchymal transition in cancer metastasis through the lymphatic system. Mol Oncol.

[76] Whyte L, Huang YY, Torres K, Mehta RG (2007). Molecular mechanisms of resveratrol action in lung cancer cells using dual protein and microarray analyses. Cancer Res.

[77] Chen JW, Tang YL, Liu H, Zhu ZY, Lu D, Geng N (2011). Anti-proliferative and anti-metastatic effects of curcumin on oral cancer cells. Hua Xi Kou Qiang Yi Xue Za Zhi.

[78] Lee SJ, Langhans SA (2012). Anaphase-promoting complex/cyclosome protein Cdc27 is a target for curcumin-induced cell cycle arrest and apoptosis. BMC Cancer.

[79] Roy D, Arason GA, Chowdhury B, Mitra A, Calaf GM (2010). Profiling of cell cycle genes of breast cells exposed to etodolac. Oncol Rep.

[80] Zhai DK, Liu B, Bai XF, Wen JA (2016). Identification of biomarkers and pathway-related modules involved in ovarian cancer based on topological centralities. J BUON.

[81] Zhou Z, He M, Shah AA, Wan Y (2016). Insights into APC/C: from cellular function to diseases and therapeutics. Cell Div.

[82] Bidkhori G, Narimani Z, Hosseini Ashtiani S, Moeini A, Nowzari-Dalini A, Masoudi-Nejad A (2013). Reconstruction of an integrated genome-scale co-expression network reveals key modules involved in lung adenocarcinoma. PLoS ONE.

[83] Kim SH, Ho JN, Jin H, Lee SC, Lee SE, Hong SK (2016). Upregulated expression of BCL2, MCM7, and CCNE1 indicate cisplatin-resistance in the set of two human bladder cancer cell lines: T24 cisplatin sensitive and T24R2 cisplatin resistant bladder cancer cell lines. Investig Clin Urol.

