# Supplementary material for: The importance of CDC27 in cancer: molecular pathology and clinical aspects
Source: Cancer Cell Int. 2021 Mar 9;21:160. doi: 10.1186/s12935-021-01860-9 (PMC7941923; doi:10.1186/s12935-021-01860-9)
Supplement: Supplementary file 1 — Additional file 1: Table S1. Protein sequence in exons and TPR motifs of CDC27. CDC27 has 19 exons with 14 TPR motifs which are located in two TPR domains. Aminoacid numbers are according to the longest isoform with 830 aminoacids. Six yellow highlighted aminoacids in brackets (at the junction of exon 8 and exon 9) are the difference between the main CDC27 isoform with 824 aminoacids and the second important functional CDC27 isoform with 830 aminoacids. Different exons are colored alternately blue and black. Red colored aminoacids at exon junctions are coded by a codon which has nucleotides on both exons. Purple highlighted aminoacids are common phosphorylation sites in the CDC27 protein which all of them are located between two TPR domains. The structure of the APC3 is consisted of 14 units of the TPR motif, which are organised as follows: dimerization domain (TPR 1 to TPR 7), IR tail binding domain (TPR 8 to TPR 11), and C-terminal domain (TPR 12 to TPR 14). [file 12935_2021_1860_MOESM1_ESM.docx]

**Table S1:** Protein sequence in exons and TPR motifs of CDC27. *CDC27* has 19 exons with 14 TPR motifs which are located in two TPR domains. Aminoacid numbers are according to the longest isoform with 830 aminoacids. Six yellow highlighted aminoacids in brackets (at the junction of exon 8 and exon 9) are the difference between the main *CDC27* isoform with 824 aminoacids and the second important functional *CDC27* isoform with 830 aminoacids. Different exons are colored alternately blue and black. Red colored aminoacids at exon junctions are coded by a codon which has nucleotides on both exons. Purple highlighted aminoacids are common phosphorylation sites in the CDC27 protein which all of them are located between two TPR domains. The structure of the APC3 is consisted of 14 units of the TPR motif, which are organised as follows: dimerization domain (TPR 1 to TPR 7), IR tail binding domain (TPR 8 to TPR 11), and C-terminal domain (TPR 12 to TPR 14).

| #Exons | Protein Sequence | #Amino-acid | TPR motifs |
| --- | --- | --- | --- |
| E1 | MTVLQEPVQ | **1-9** | **TPR1**: 5-34 |
| E2 | AAIWQALNHYAYRDAVFLAERLYAEV | **10-35** |  |
| E3 | HSEEALFLLATCYYRSGKAYKAYRLLKGHSCTTPQCKYLLAKCCVDLSK | **36-84** | **TPR2**: 38-63  **TPR3**: 69-93  **TPR4**:114-144  **TPR5:** 149-167 |
| E4 | LAEGEQILSGGVFNKQKSHDDIVTEFGDSACFTLSLLGHVYC | **85-126** |  |
| E5 | KTDRLAKGSECYQKSLSLNPFLWSPFESLCEIG | **127-159** |  |
| E6 | EKPDPDQTFKFTSLQNFSNCLPNSCTTQVPNHSLSHRQPETVLTETPQDTI | **160-210** |  |
| E7 | ELNRLNLESSNSKYSLNTDSSVSYIDSAVISPDTV  PLGTGT*SILSKQVQNKPKTGR*SLLGGPAALSPLTPS | **211-281** |  |
| E8 | FGILPLETPSPGDGSYLQNYTNTPPVIDVPSTGAPSK  [K] | **282-318**  **319** |  |
| E9 | [TFRVL]  QSVARIGQTGTKSVFSQSGNSREVTPILAQTQSSGPQTS | **320-324**  **325-363** |  |
| E10 | TTPQVLSPTITSPPNALPRRSSRLFTSDSSTTK | **364-396** |  |
| E11 | ENSKKLKMKFPPKIPNRKTKSKTNKGGITQPNIND  SLEITKLDSSIISEGKISTITPQIQAFNLQKAAAE | **397-466** | **TPR6**: 461-496  **TPR7**: 505-535  **TPR8**: 541-552  **TPR9**: 573-602  **TPR10**: 609-636  **TPR11**:641-670  **TPR12**:674-707  **TPR13**:709-738  **TPR14**:743-772 |
| E12 | GLMSLLREMGKGYLALCSYNCKEAINILSHLPSHHYNTGWVLCQIGRAYFELSEYMQ | **467-523** |  |
| E13 | AERIFSEVRRIENYR  VEGMEIYSTTLWHLQKDVALSVLSKDLTDMDKNSPE | **524-574** |  |
| E14 | AWCAAGNCFSLQREHDIAIKFFQRAIQVDPNY  AYAYTLLGHEFVLTEELDKALACFRNAIRVNPRHYNAW | **575-644** |  |
| E15 | YGLGMIYYKQEKFSLAEMHFQKALDINPQSSVLLCHIGV | **645-683** |  |
| E16 | VQHALKKSEKALDTLNKAIVIDPKNPLCKFHRASVLFANEKYK | **684-726** |  |
| E17 | SALQELEELKQIVPKESLVYFLIGK | **727-751** |  |
| E18 | VYKKLGQTHLALMNFSWAMDLDPKGANNQIKEAIDKRYLPDDEEPITQEEQIM | **752-804** |  |
| E19 | GTDESQESSMTDADDTQLHAAESDEF | **805-830** |  |
