# Supplementary figures and images for: The importance of CDC27 in cancer: molecular pathology and clinical aspects
Source: Cancer Cell Int. 2021 Mar 9;21:160. doi: 10.1186/s12935-021-01860-9 (PMC7941923; doi:10.1186/s12935-021-01860-9)

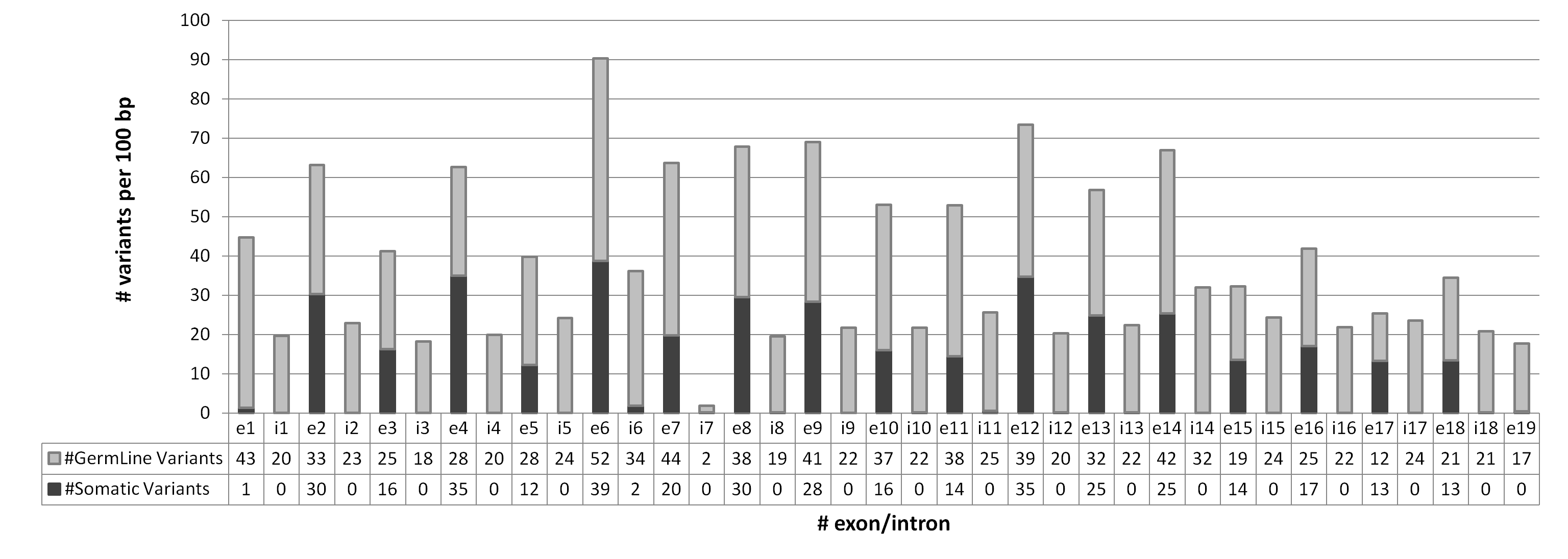

Supplement: Supplementary file 2 — Additional file 2: Figure S1. The frequency of potentially somatic and germline variants at exons and introns in CDC27 gene. The frequency is calculated as the number of variants per 100 bases in each exon or intron (Number of variants is divided to the exon or intron length and then multiplied by 100). About 588 CDC27 variants were listed in COSMIC (554 variants on exons). This means that potentially somatic cancer variants may compose more than 25% of detected variants on CDC27 exons. [file 12935_2021_1860_MOESM2_ESM.tif]
